# The Dental Register of the West

**Published:** 1848-01

**Authors:** 


*The Dental Register of the West.*—We have received the first number of a periodical published under the above title. It contains fifty-four pages, and is published by the order of the Mississippi Valley Association of Dental Surgeons, and edited by professor James Taylor, M. D of

Cincinnati, Ohio, and B. B. Brown, M. D. of St. Louis, Mo. The editors are both well known to the profession as dentists of high standing, and under their management there need be no fear with regard to the manner in which it will be conducted. It is to be published quarterly at $2 per annum. Besides the proceedings of the last meeting of the Association, whose organ it is, it contains several valuable and highly interesting articles. We commend it to the patronage of the profession, and most cordially wish our brethren of the west success in so commendable an enterprise.—*Balt. Ed.*

